# Friend or Foe? Implication of the autophagy-lysosome pathway in SARS-CoV-2 infection and COVID-19

**DOI:** 10.7150/ijbs.72544

**Published:** 2022-07-11

**Authors:** Weifeng He, Yuan Gao, Jing Zhou, Yi Shi, Dajing Xia, Han-Ming Shen

**Affiliations:** 1 State Key Laboratory of Trauma, Burn and Combined Injury, Institute of Burn Research, Southwest Hospital, Army Medical University, Chongqing, China; 2 Faculty of Health Sciences, University of Macau, Macau, China; 3 Chinese Academy of Sciences Key Laboratory of Pathogenic Microbiology and Immunology, Institute of Microbiology, Chinese Academy of Sciences, 100101 Beijing, China; 4 Department of Physiology, School of Preclinical Medicine, Guangxi Medical University, Nanning, Guangxi Province, China; 5 Department of Toxicology of School of Public Health, Department of Gynecologic Oncology of Women's Hospital; Department of Central Laboratory, Affiliated Hangzhou First People's Hospital, Zhejiang University School of Medicine, Hangzhou, Zhejiang, China

**Keywords:** SARS-CoV-2, COVID-19, autophagy, lysosome, clinical trials, therapeutics

## Abstract

There is increasing amount of evidence indicating the close interplays between the replication cycle of SARS-CoV-2 and the autophagy-lysosome pathway in the host cells. While autophagy machinery is known to either assist or inhibit the viral replication process, the reciprocal effects of the SARS-CoV-2 on the autophagy-lysosome pathway have also been increasingly appreciated. More importantly, despite the disappointing results from the clinical trials of chloroquine and hydroxychloroquine in treatment of COVID-19, there is still ongoing effort in discovering new therapeutics targeting the autophagy-lysosome pathway. In this review, we provide an update-to-date summary of the interplays between the autophagy-lysosome pathway in the host cells and the pathogen SARS-CoV-2 at the molecular level, to highlight the prognostic value of autophagy markers in COVID-19 patients and to discuss the potential of developing novel therapeutic strategies for COVID-19 by targeting the autophagy-lysosome pathway. Thus, understanding the nature of such interactions between SARS-CoV-2 and the autophagy-lysosome pathway in the host cells is expected to provide novel strategies in battling against this global pandemic.

## Introduction

Coronaviruses (CoVs) are a group of RNA viruses with a positive-sense single-stranded RNA genome. In the last two decades, there are three major CoVs that caused severe diseases in humans: first, the severe acute respiratory syndrome coronavirus (SARS-CoV), then the Middle East respiratory syndrome coronavirus (MERS-CoV), and now the severe acute respiratory syndrome coronavirus 2 (SARS-CoV-2), the causative agent of the ongoing coronavirus disease of 2019 (COVID-19) pandemic. Since the outbreak occurred at the end of 2019, SARS-CoV-2 has quickly spread to more than 200 countries and regions around the globe. Up to date, based on WHO statistics, there have been more than 500 million cumulative cases of COVID-19, with more than 6.2 million deaths worldwide (https://covid19.who.int/). At present, quarantine-isolation-social distance and vaccination remain as the main strategies in control of this pandemic, while searching for effective therapy remains as a major challenge 1-3.

**Figure 1:**
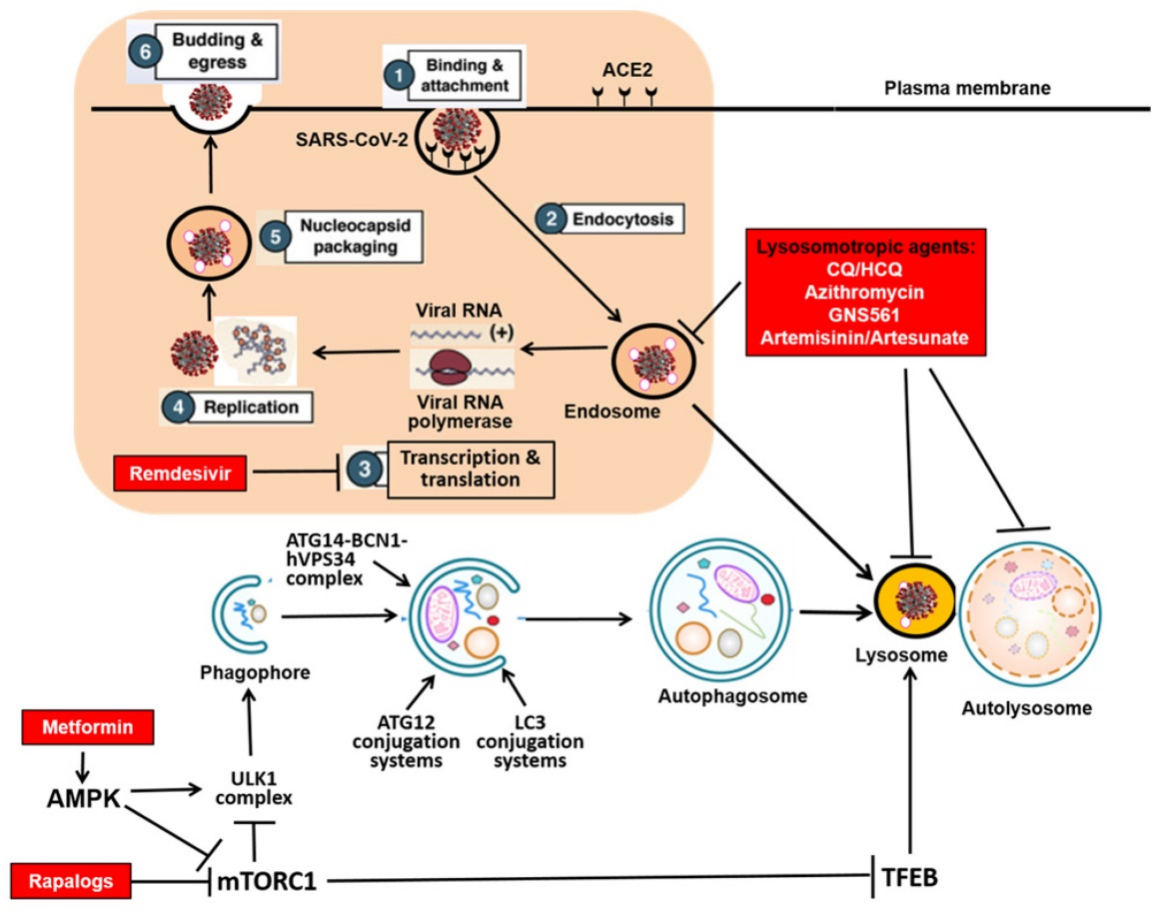
** SARS-CoV-2 replication cycle and the autophagy-lysosome system in the host cells.** The shielded area shows the 6 steps of the viral replication cycle of SARS-CoV-2, and the link with the autophagy-lysosome pathway. The red boxes indicate the therapeutics under development (in clinical trials) against COVID-19 with relevance to the autophagy- lysosome pathway.

The SARS-CoV-2 genome encodes 11 genes with 14 open reading frames (ORFs) that produce 16 nonstructural proteins (NSP1-NSP16), 4 structural proteins (spike protein-S, membrane protein-M, nucleocapsid protein-N, and envelope protein-E), and 9 accessory proteins (ORF3a, ORF3b, ORF6, ORF7a, ORF7b, ORF8, ORF9b, ORF9c and ORF10)^4^. One key aspect is the understanding of the viral replication process, consisting of the following 6 steps 5, 6 (**Figure 1**): (i) Binding and attachment: entry of the virus into the host cells *via* the interaction between the viral S protein and the host receptor, angiotensin converting enzyme-2 (ACE2); (ii) Endocytosis: Fusion of viral membrane with the host cell membrane via S protein cleavage mediated by the host serine proteases in the host endocytic pathway (endosomes and lysosomes); (iii) Transcription and translation of viral proteins from the viral RNAs; (iv) Viral replication within the replicative membranous compartment; (v) Nucleocapsid packaging: Virus assembly and package at the endoplasmic reticulum (ER) and/or the Golgi complex; and finally (vi) Budding and egress: Release of new virions *via* exocytosis. Understanding such a replication process and the interaction of the viral proteome with the host proteome is the foundation for development of effective interventional and therapeutic approaches and agents for control of this deadly pandemic. For instance, remdesivir, the FDA-approved drug for COVID-19, is a specific inhibitor for the RNA-dependent RNA polymerase to block the Step 3 described above 7, 8 (**Figure 1**). In search of the molecular mechanisms in control of viral replication, the involvement of the autophagy-lysosome pathway in the host cells has attracted substantial attention. It is now believed that this pathway is closely implicated in several steps of viral replication, including Step 1 (entry), Step 2 (endocytosis), Step 3 (transcription and translation), as well as Step 6 (exocytosis) 9-12.

Autophagy is an evolutionarily conserved catabolic process in which the intracellular components such as protein aggregates and damaged organelles are delivered to lysosome for degradation 13. In mammalian cells, there are 3 types of autophagy: macroautophagy, chaperon-mediated autophagy (CMA) and microautophagy. Among them, macroautophagy or autophagy is featured with formation of the double membrane structure called autophagosome, a process controlled by a group of proteins encoded by autophagy-related-genes (*ATGs*) 14. As shown in **Figure 1,** the autophagy process consists of several consecutive stages, including (i) induction or initiation, (ii) nucleation/expansion/elongation, and (iii) maturation/fusion/degradation. Specifically, the induction or initiation stage is controlled by the ULK1 complex directly downstream of upstream kinases such as mammalian target of rapamycin complex 1 (mTORC1) and AMPK protein kinase (AMPK). The nucleation/expansion/elongation is mediated by the ATG14-BCN1-hVPS34/class III phosphatidylinositol 3-kinases (PI3K) complex, as well as the two ubiquitin-like conjugation systems (ATG12 and LC3). Finally, the last stage of maturation/fusion/degradation refers to the process in which the completed autophagosome fuses with lysosome to form autolysosome where the luminal contents/cargos are degraded. Depending on the nature of the cargos, autophagy is categorized into non-selective and selective types and the latter includes specific cargos such mitochondria (mitophagy), endoplasmic reticulum (ER) (ER-phagy), protein aggregates (aggrephagy), invaded pathogens (xenophagy), etc. 15-18. At present, the biological functions of autophagy have been extensively studied. Autophagy plays an important role in various physiological and pathological processes, including cell survival, cell death, aging, immunity and metabolism 19, 20. More importantly, autophagy has been implicated in the pathogenesis of many human diseases, such as cancer, neurodegenerative diseases, metabolic disorders, as well as immunity and infection 21.

In modern cell biology, especially with the emerging research on autophagy, the importance of lysosome has been increasingly appreciated. First, lysosome is the key organelle responsible for degradation of cargos delivered *via* various forms of autophagy as well as *via* the endocytic pathway 22, 23. Second, lysosome serves as the signaling nod for intracellular signaling pathways mediated by nutrients. One good example is amino acids-mediated mTORC1 lysosomal localization and activation 24. Third, lysosome is closely associated with many other cellular processes, including exocytosis, plasma membrane repair, defense against pathogens, and cell death 23, 25, 26. Therefore, autophagy and lysosome constitute an important system in maintaining the cellular homeostasis. Since the outbreak of COVID-19, a great deal of effort has been attributed to understand the potential role of the autophagy-lysosome pathway in the pathogenicity of SARS-CoV-2 and the disease caused, COVID-19. The phenomenal rise and fall of chloroquine (CQ)/hydroxychloroquine (HCQ) in treatment of COVID-19 added the fuel into this hot area of research. So far, a number of reviews, commentaries and hypotheses have already been published with extensive discussion on the relevant topics 9-12, 27-31. However, it still remains largely controversial regarding the exact role of the autophagy-lysosome pathway in COVID-19 and whether the drugs targeting this pathway such as CQ/HCQ is effective in treatment of COVID-19. In this review, we attempt to summarize the latest findings on the interplays between SARS-CoV-2 and the autophagy-lysosome pathway in the host cells, and discuss the therapeutic potentials of compounds (other than CQ/HCQ) that target the autophagy-lysosome pathway, in particular those in clinical trials. Understanding the nature of such interactions between of SARS-CoV-2 and the autophagy-lysosome pathway in the host cells is expected to provide novel strategies in battling against this deadly pandemic.

## 2. Effect of the autophagy-lysosome pathway on SARS-CoV-2

Once entering the host cells, SARS-CoV-2 is expected to elicit wide ranges responses from the host machinery which would impact the viral replication. A recent study conducted a profiling analysis of miRNA and mRNA expression in patients with SARS-CoV-2 infection and found an extensive autophagy interaction network in cells infected with SARS-CoV-2 and revealed several novel autophagy-related biomarkers 32. Such analysis thus provides a valuable source of information to further dissect the effect of the autophagy-lysosome on SARS-CoV-2, and vice versa.

### 2.1 The pro-viral function

**Figure 2:**
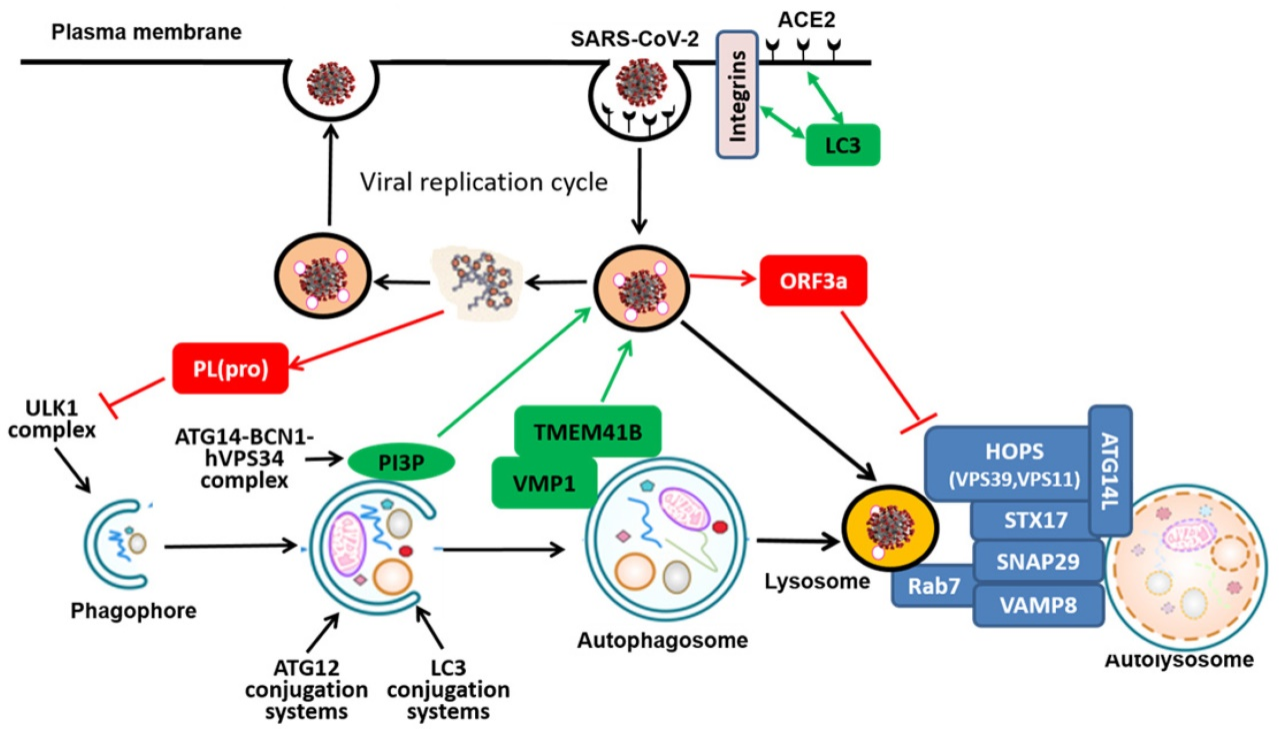
** Molecular mechanisms mediating the interplays between the SARS-CoV-2 replication cycle and the autophagy-lysosome pathway in the host cells.** The effects of the autophagy-lysosome pathway on the viral replication cycle are shown in green and the reciprocal effects of viral proteins on the autophagy-lysosome pathway are shown in red

At present, there is evidence indicating part of the autophagy machinery might participate in the replication process of SARS-CoV-2, thus serving as a pro-viral mechanism. ACE2 and integrins are the most well-recognized receptor and co-receptors, respectively, for CoV infection, via direct binding to the receptor-binding domain (RBD) of the spike protein of SARS-CoV-2 33, 34. Two recent studies independently identified the presence of LC3-interacting region (LIR) motifs in the tails of ACE2 and integrin β3 35, 36 (**Figure 2**). Although the exact functional implication of this LIR remains to be further investigated, it is speculated that the presence of LIR in ACE2 and integrin β3 is likely to assist the viral entry and replication in the host cells.

After binding to its cell surface receptor ACE2, the SARS-CoV-2 virus enters the host cell through directly fusing with plasma membrane (the cell surface pathway) or undergoing endocytosis via endosome/lysosome (the endocytic pathway)^37^, ^38^. For the second pathway, parts of internalized receptors are retrieved to recycling endosomes by which they will travel back to plasma membrane, while others are delivered to late endosomes-lysosome for degradation 39. SNX27, one of the sorting nexin (SNX) family members, is known to regulate the trafficking of the endosomal receptors from the early endosomes to recycling endosomes 40-42. Recent studies have studied the complex structure of ACE2 and SNX27 and found that SNX27 could regulate the abundance of ACE2 on the cell surface 43. On the other hand, SNX27 directs the trafficking of ACE2/virus, voiding lysosome/late endosome entry of the virus in Huh7 cells 43. Such findings thus point to SNX27 as a new regulator of ACE2 trafficking and viral infection, implying that SNX27-retromer could be used as an antiviral therapeutic target.

In addition to ACE2 and integrin locating at the plasma membrane, other membrane proteins residing at intracellular organelles such as ER and Golgi are also known to be implicated in the viral replication process 44. Transmembrane protein 41B (TMEM41B) is an ER-localized transmembrane protein with relatively little known function, while vacuole membrane protein 1 (VMP1) is relatively well known to be implicated in the autophagy process 45-47. Recent work from 3 research groups by using genome-wide CRISPR-Cas9 screening have independently identified TMEM41B as a novel autophagy regulator in conjunction with VMP1 48-50: TMEM41B and VMP1 directly interact with each other *via* their characteristic transmembrane domain and are required for autophagosome (**Figure 2**). Interestingly, two very recent studies used full-genome loss of function CRISPR-Cas9 screening and independently found and validated that TMEM41B is required for SARS-CoV-2 infection 51, 52. In their studies, deletion of *TMEM41B* markedly reduced infectivity, which could be fully restored with the reconstitution of *TMEM41B.* Functionally, TMEM41B interacts with VMP1 in the CoV infection, similar to the autophagy process as described earlier. Similarly, another transmembrane protein TMEM106B is also found to be involved in SARS-CoV-2 infection 53. In addition to those mentioned above, recent work by Ou et al. investigated the importance of Cathepsin L (CatL), a protease responsible for recycling cellular proteins inside lysosomes, for the SARS-CoV-2 entry into the host cell and concluded that the inhibition of CatL decreases virus entry 38, 54. Such findings thus provide clues that link the viral replication of SARS-CoV-2 with the autophagy-lysosome pathway and those common molecules may serve as the targets for intervention of both processes.

Intriguingly, canonical autophagy proteins such as BECN1, ATG5, and ATG7 are not required for the viral infection of SARS-CoV-2 in the host cells 51; and TMEM41B is the only gene implicated in autophagy that scores as a significant hit in cells infected with SARS-CoV-2 52. Apparently, more work is needed to further illustrate how TMEM41B and VMP1 differentiate the upstream signaling from viral infection and autophagy inducers, and designate their distinctive functions under different stress conditions.

It is known that part of the viral entry mechanisms of SARS-CoV-2 heavily involves cellular membrane structure and lipid biosynthetic pathways 55, 56. The class III PI3K (phosphoinositide 3-kinase) or Vps34 (vacuolar protein sorting 34) is an evolutionarily conserved lipid kinase in producing PI3P (phosphatidylinositol 3-phosphate), which plays critical roles in a variety of cellular processes including autophagy 57, 58. PI3K is activated in cells infected with SARS-CoV-2 to produce PI3P while inhibition or depletion of PI3K reduces rival replication and viral DMV formation 59. Moreover, Silvas et al. found that inhibition of VPS34 kinase activity by VPS34-IN1, a well-defined specific small molecule inhibitor, led to suppression of SARS-CoV-2 replication in human airway epithelial cells 60. Such results indicate the possibility that the product of VPS34, PI3P, is implicated in the viral replication process (**Figure 2**). However, it was not determined in their study whether the anti-viral activity of VPS34 inhibitors is mediated via suppression of autophagy. Correspondingly, Yuen et al. utilized a number of autophagy inhibitors including the ULK1 inhibitor SBI0206965, VPS34 inhibitor VPS34-IN1, pan PI3K inhibitor 3-MA, as well as the lysosome inhibitor HCQ and found that among them, only the VPS34 inhibitor VPS34-IN1 is effective in suppressing SARS-CoV-2 replication in both *in vitro* and *ex vivo* models 61. Since VPS34 and its main product PI3P are well known to have multiple functions such as in regulation of endocytic trafficking, there is a possibility that VPS34 may participate the replication process of SARS-CoV-2 independent of its function in autophagy. Thus, the above mentioned data also support the notion that VPS34 inhibitors but not autophagy inhibitors *per se* may have the therapeutic potential against COVID-19.

### 2.2 The anti-viral function

In contrast to the pro-viral function of some the autophagy-related proteins as discussed above, the anti-viral function against SARS-CoV-2 by autophagy remains largely circumstantial. ATG16L1 is one of the key ATGs in control of autophagosome biogenesis* via* the formation of the ATG5-ATG12-ATG16L1 complex in mediating LC3 lipidation 62. It has been reported that ATG16L1 risk allele (ATG16L1-T300A) was associated with increased risk for Crohn's disease (CD), probably due to impaired autophagy/xenophagy and reduced ability in clearing pathogens 63. The ATG16L1 risk allele T300 was mainly found in non-European, particularly African descents, while the ATG16L1 A300 allele was predominantly found in Europeans. Consistently, HCT116 cells with ATG16L1 T300 expressed greater amounts of GRP78 and SARS-CoV-2 receptor ACE2 compared to cells with the ATG16L1 A300. Such results thus suggest that ATG16L1 T300 expression might be associated with a greater ability to be infected and to reproduce SARS-CoV-2 64. However, it has not been determined whether the increased risk of SARS-CoV-2 infection is causatively linked to impaired autophagy in those subjects with the ATG16L1 T300 allele.

## 3. The reciprocal effect of SARS-CoV-2 on the autophagy-lysosome pathway in the host cells

Similar to SARS-CoVs and MERS-CoVs, once entering the host cells, SARS-CoV-2 will impact various cellular processes in the host cells reciprocally. Among them, the autophagy-lysosome pathway appears to be particularly relevant and important. In theory, SARS-CoV-2 may either activate or inhibit the autophagy process, similar to the effect of other CoVs (SARS-CoVs and MERS-CoVs). By modelling the putative virus-host interplays *via* a combination of computational and knowledgebase with experimentally validated host interactome proteins and differentially expressed host genes in SARS-CoV-2 infection, autophagy signaling pathway has emerged as one of the key processes, among several other pathways such as HIF-1 signaling, RIG-1 signaling, etc. 65. Gassen et al. reported impaired autophagy in both an animal model and in COVID-19 patients, evidenced by reduced expression of proteins implicated in various stages of autophagy 66. In the section below, we mainly present and discuss the regulatory effects of SARS-CoV-2 on the autophagy-lysosome pathway in the host cells.

### 3.1 Suppression of autophagy at the early stage

As introduced above, the early stage of autophagy includes several sequential events leading to the formation of autophagosome, which is controlled by several ATG complexes. It has been reported recently that expression of papain-like protease PL(pro), a viral protease from SARS-CoV-2, is sufficient to impair starvation-induced autophagy, possibly via reduction of ULK1 protein level and disrupt formation of ULK1-ATG13 complex 67 (**Figure 2**). A systematic analysis of a wide range of SARS-CoV-2 proteins on autophagy identified several individual viral proteins that target different stages of autophagy: both ORF3a and ORF7a prevent the fusion between autophagosomes and lysosomes, whereas Nsp15 indeed impairs formation of autophagosomes 68. Intriguingly, such observations were inconsistent with a previous report in which viral membrane-associated papain-like protease PLP2 (PLP2-TM) from one CoV (HCoV-NL63) was found to interact with key ATGs such as LC3 and BCN1 to promote the early stage of autophagy 69. Such discrepancy is possibly caused by different CoVs and cell models used and it is also not known how the inhibition (or activation) of autophagy would affect the viral replication in the host cells.

### 3.2 Suppression of autophagy at the late stage

At the late stage of autophagy involves the fusion of autophagosome with lysosome, a process known to be controlled by several protein complexes including SNARE (STX17-SNAP29-VAMP8), HOPS (homotypic fusion and protein sorting) (VPS39, VPS11) and ATGs (ATG14) 22, 70 (**Figure 2**). So far, there are several independent studies demonstrating that ORF3a, an accessory viral protein from SARS-CoV-2, can suppress the late stage of autophagy 71-75. For instance, Miao et al. recently reported that overexpression of ORF3a inhibits autophagy flux at the late stage 72. Mechanistically, ORF3a located at the late endosome is capable of directly binding to VPS39, an important component of the HOPS complex that is known to be implicated in autophagosome-lysosome fusion via direct interaction with STX17 76, 77. Intriguingly, the same ORF3a from SARS-CoV did not have the same function, suggesting that the inhibitory effect on the late stage of autophagy is specific to SARS-CoV-2. Moreover, the interaction between ORF3a and VPS39 was also found in an unbiased screening study 78. In their study, the authors cloned, tagged and expressed 26 of the 29 SARS-CoV-2 proteins in human cells and then identified the interactome of SARS-CoV-2 with host proteins 78. By using affinity-purification mass spectrometry approach, they identified more than 300 human proteins that physically associated with the SARS-CoV-2 proteins. Among them, the viral protein OFR3a was found to interact with VPS39 and VPS11, two important components of the HOPS complex 78. Similar results were also found when *Drosophila* were used an *in vivo* model to characterize the SARS-CoV-2 pathogenicity, and found that overexpression of ORF3a is capable of inducing apoptosis and inflammation in the nervous system 73. Importantly, such effects of ORF3a could be inhibited by CQ, suggesting the possible involvement of the autophagy-lysosome pathway, although no direct evidence was provided 73. The consistent results on the role of OFR3a and HOPS complex from different studies support the notion that specific viral proteins from CoVs including SARS-CoV-2 such as ORF3a possess the ability of suppressing autophagy at the late stage of autophagy.

Meanwhile, Sun et al. recently found a viral protein NSP6 of SARS-CoV-2 might impair the autophagic flux at the late stage to active inflammasomes by targeting ATP6AP1 79. In their study, enforced expression of NSP6 induced the concomitant accumulation of LC3B-II and SQSTM1/p62 in lung epithelial cells, suggesting that NSP6 mainly affects lysosome acidification but not autophagosome-lysosome fusion 79. Mechanistically, NSP6 impairs lysosome acidification by interacting with ATP6AP1 to block the pro-cathepsin D cleavage-mediated activation 79.

In addition to the negative effects of proteins derived from SARS-CoV-2 on the autophagy machinery as discussed above, a recent report demonstrated that CoVs such as MHV are transported to and enriched in late endosome and lysosome 80. Functionally, enrichment of the virus in the endocytic organelles leads to lysosome deacidification, inactivation of lysosomal digestive enzymes, and disruption of antigen presentation, and such changes are associated with the viral unconventional egress, a lysosome-dependent exocytosis process controlled by Arf-like small GTPase Arl8b and RAB7 80. At present, it is not known whether the accumulation of SARS-CoV-2 in endosome and lysosome would have the similar functional impact. Nevertheless, the close interplay between the autophagosome-lysosome pathway and the viral replication process makes the endosome-lysosome a legitimate target for development of therapeutic approaches against SARS-CoV-2 and COVID-19.

### 3.3 Activation of the autophagy-lysosome system by SARS-CoV-2

Although majority of reports found the inhibitory effects of SARS-CoV-2 proteins on the autophagy-lysosome system, as discussed above, there is evidence showing the positive regulatory effects. For instance, SARS-CoV-2 ORF3a is capable of prompting the lysosomal function and the exocytosis process to facilitate the viral egress 81. Lysosome-Associated Membrane Protein-2a (LAMP2a) is known to be a key player in chaperone-mediated autophagy (CMA) 82. A recent study performed an RNA-protein interaction analysis of SARS-CoV-2 5'-and 3'-untranslated regions and found enhanced LAMP2a expression and upregulated autophagic flux in cells infected with SARS-CoV-2 83. Moreover, the positive regulatory acitivity of SARS-CoV-2 is functionally relevant to the pathogenesis of COVID-19. For instance, ORF8 of SARS-CoV-2 directly interacts with MHC-Ι and mediates its down-regulation and suppression of a main antigen presenting system *via* promoting the autophagy-lysosome pathway 84. The autophagy-promoting activity of SARS-CoV-2 has also been demonstrated in animal models in vivo, including the long-tailed or crab-eating macaque (Macaca fascicularis), human angiotensin-converting enzyme 2 (hACE2) transgenic mice, and xenografted human lung tissues 85. Intriguingly, while SARS-CoV-2 induces autophagosome formation, it also blocks autophagosome-lysosome fusion 85. Nevertheless, such results thus provide an experimental basis for using lysosomotropic agents including chloroquine for treatment of COVID-19, a topic still under extensive debate 31.

## 4. Changes of the autophagy-lysosome as biomarkers for prognosis of COVID-19 patients

With the persistence the COVID-19, it becomes more feasible to access to COVID-19 patients' biospecimen for analysis of bio-markers related to autophagy-lysosome. Indeed, such analyses have provided insightful information for a better understanding of the implication of autophagy-lysosome in COVID-19. For instance, Fang and colleagues have performed a transcriptomic analysis in peripheral blood mononuclear cells (PBMCs) from COVID-19 patients. By doing so, they identified the aberrant upregulation of genes in the lysosome pathway 86. In particular, a decrease in LC3B and p62 levels in patients' sera was associated with moderate-to-severe COVID-19, thus suggesting that such biomarkers might be used to stratify COVID-19 patients with different severity 86. Such findings are generally consistent with a recent report in which the authors performed network analysis and transcriptome profiling in human cells infected with SARS-CoV-2 *in vitro* and in human samples of COVID-19 patients 87. In their study, SARS-CoV-2 infection led to down-regulation of genes related to autophagy and lysosomal function, a possible factor contributing to increased viral replication 87. Similar results were also found in another report in which the authors compared about 140 parameters of cellular and humoral immune markers in both COVID-19 patients and healthy controls, with integration of about 30 common clinical and laboratory parameters 88. Notably, a lower expression level of autophagy genes was correlated with severity of COVID-19, thus providing another piece of clinical evidence linking autophagy to the pathogenesis of COVID-19. Intriguingly, a recent study found a higher level of serum Beclin1 in COVID-19 patients than the healthy controls and proposed that serum Beclin1 has a predictive value in assessing the disease severity of COVID-19 89. Taken together, it appears that alteration of the autophagy-lysosome pathway is a common phenomenon in COVID-19 patients and changes of autophagy-related biomarkers could be useful for prognosis of COVID-19 patients. Such findings in fact provide some clues for modulating the autophagy-lysosome pathway as a therapeutic strategy in combating COVID-19, to be discussed in details below.

## 5. Therapeutic potential of autophagy-lysosome modulators in COVID-19

At present, one urgent unmet medical need for the treatment of COVID-19 is the lack of specific therapeutic drugs. Remdesivir is the only FDA-approved therapeutic agent so far, while its efficacy against COVID-19 remains inconsistent and controversial 7, 90 and WHO recommends against the use of remdesivir in COVID-19 patients (https://www.who.int/news-room/feature-stories/detail/who-recommends-against-the-use-of-remdesivir-in-covid-19-patients). On the other hand, the fanfare of CQ/HCQ in treatment of COVID-19 has attracted tremendous amount of public attention, partly due to some political reasons. At present, despite the strong evidence from preclinical studies (cell culture and animal models), the outcomes of clinical trials using CQ/HCQ are largely disappointing, and FDA has revoked its authorization for the emergency use of CQ/HCQ in COVID-19 patients (https://www.fda.gov/news-events/press-announcements/coronavirus-covid-19-update-fda-revokes-emergency-use-authorization-chloroquine-and). Since the use of CQ/HCQ in COVID-19 has been extensively discussed 91-93, in this review we intend to only cover other potential therapeutics targeting the autophagy-lysosome pathway in the host cells. Some of the relevant therapeutic agents are highlighted in **Figure 1** showing their putative targets in the autophagy-lysosome pathway. A very recent report used a biological activity-based modeling (BABM) approach to predict compounds with a potential activity against SARS-CoV-2 94. Interestingly, among more than 300 predicted compounds, viral entry and autophagy were found to be the two main targets 94, thus clearly supporting the notion that the autophagy could serve as a legitimate target for anti-viral therapy against SARS-CoV-2. Moreover, Zhu and colleagues made a similar attempt by a systematic analysis of their in-house database to compare drug activity profiles in a cytopathic effect (CPE) assay of SARS-CoV-2 95. Through analysis of nearly 1000 assays, they found that the activity profiles of the autophagy and the AP-1 signaling pathway are significantly correlated with the anti-SARS-CoV-2 activity profile^95^, thus once again highlights the relevance of autophagy in the search of effective therapeutics against COVID-19.

### 5.1 Lysosomotropic agents

At present, there are still active efforts in discovery of novel compounds targeting the endosome-lysosome pathway. Among them, compounds with a lysosomotropic property appears to be particularly attractive, based on the notion that targeting the cellular acidic organelles (endosomes and lysosomes) that contains the endocytic viruses have potential therapeutic effects against SARS-CoV-2 and for the treatment of COVID-19 96, 97. So it is not surprising that even at this stage, there are more than 100 clinical trials ongoing world-wide to test the therapeutic efficacy of CQ/HCQ in COVID-19 (**Table 1**). Azithromycin, a commonly used wide spectrum antibiotic for treatment of a number of bacterial infections, has been shown to possess direct and indirect antiviral activity in multiple systems 98. Pharmacologically, Azithromycin is known to accumulate inside lysosomes and its lysosomotropic property is related to its anti-bacterial and anti-viral function 99, 100. Recent results from *in vitro* studies demonstrated the anti-SARS-CoV-2 activity of Azithromycin 101-103. For instance, Azithromycin is able to block the entry of SARS-CoV-2 in HEK293T-ACE2 and Caco2 cells *via* disruption the fusion process between viral and vacuolar membranes, a process related to its lysosomotropic property 103. Moreover, there are numerous reports on clinical trials, using either Azithromycin alone or in combination with CQ/HCQ in treatment of COVID-19, and the results have been well summarized 104-108. Generally, there is evidence showing the therapeutic benefits of using Azithromycin alone or in combination with CQ/HCQ in treatment of COVID-19. Apparently, more work is needed, including mechanistic understanding on the anti-viral effects of Azithromycin against SARS-CoV-2. At present, there are several dozen of clinical trials still ongoing, either using Azithromycin alone or in combination with CQ/HCQ (**Table 1**), including randomized double blind placebo control clinical trials to prove the therapeutic efficacy.

GNS561 is a small lipophilic molecule with a lysosomotropic property that is capable of neutralizing lysosomal pH and inhibit late-stage autophagy and it is currently under development as a therapeutic agent for liver cancer 109. As the effect of GNS561 is similar to the effect of CQ/HCQ, Halfon et al. reported that it has a potent antiviral effect against two SARS-CoV-2 strains, with even a higher efficacy than that of CQ 110. There is one ongoing clinical trial to test its therapeutic efficacy in COVID-19 (**Table 1**).

Recently, Gorshkov K et al. reported several compounds (*ie*. ROC-325, clomipramine, and hycanthone) that are capable of blocking the cytopathic effect of SARS-CoV-2 in Vero E6 cells 111. Their anti-viral efficacy was found to be similar to that of remdesivir, the specific inhibitor of viral RNA-dependent RNA polymerase of CoVs and at present it is the only FDA-approved COVID-19 drug 7, 112. Mechanistically these compounds increased lysosomal pH, blocked lysosome functioning and autophagy, prevented viral particle entry and eventually reduced the viral replication ability 111. It remains to be tested whether remdesivir also has similar effects on endosome and lysosome.

Due to the urgency of the SARS-CoV-2 pandemic, one common approach for fast-track development of therapeutics is to repurpose the current anti-viral and anti-bacterial drugs for treatment of COVID-19. For instance, Tilorone, Quinacrine and Pyronaridine have been repurposed from Ebola and Marburg Virus Inhibitors. They are known to have lysosomotropic property and capable of suppressing SARS-CoV-2 replication at a nM level at cell culture *in vitro* 113, thus justifying further investigation using *in vivo* animal models and even in clinical trials.

Among the repurposed drugs for COVID-19, artemisinin and its related compounds have received particular attention. Artemisinin is from the extracts of the medicinal plant, *Artemisia annua* L., which has been widely used as an antimalarial drug. So far one of the assumptions that may explain the less-than-expected severity of pandemic of COVID-19 in Africa, and particularly in malaria endemic areas, could be the use of antimalarial drugs, specifically, the artemisinin-based combination therapy 114. Several studies have demonstrated that artemisinin and some of its derivatives such as artesunate, artemether, and dihydroartemisinin are capable of inhibiting SARS-CoV-2 replication *in vitro* 115-117. Importantly, the effective concentrations of these compounds *in vitro* are within the range of clinically achievable level in plasma after administration in patients 116, 117. The mechanisms underlying the anti-viral activity are still under investigation. On one hand, artesunate, artemisinin and artenimol have been shown to interact with Lys353 and Lys31 binding hotspots of the S protein of SARS-CoV-2 118, indicating that they act *via* suppression of viral entry. On the other hand, it was also found that artemisinin likely acts at the post-entry stages of viral infection since it had minimal effects on infection of Vero E6 or Calu-3 cells by a reporter virus pseudo-typed by the SARS-CoV-2 spike protein 115. Such discrepancy is possibly caused by the different methods (full virus *vs* single S protein). Interestingly, one earlier study from our lab showed that artesunate has a lysosomotropic property with enrichment in lysosome and is able to activate lysosomal function 119, thus raising the possibility that such lysosome-related effects are related to its anti-viral activity. At present, there are numerous clinical trials ongoing to test the therapeutic efficacy of artesunate and artemisinin, either alone or in combination with other anti-viral and anti-inflammatory agents (**Table 1**). A clinical trial in China (not registered at NIH Clinical Trial website) have reported that 43 cases of confirmed COVID-19 patients artesunate can shorten the treatment time of COVID-19, improve prognosis and eliminate pathogens, with fewer adverse reactions and a good application prospect 120. Therefore, results from the ongoing clinical trials will be valuable to enhance our weaponary in the battle against COVID-19, especially for many developing contries in Africa.

In addition to artemisinin, the pharmacological properties of several other natural compounds have gained increasing attention as a possible alternative therapy in COVID-19. In particular, several naturally-occurring herbal compounds (mostly polyphenols) are reported to produce widespread antiviral, anti-inflammatory, and anti-oxidant effects and partly via modulation of autophagy 121. For instance, Resveratrol, a well-known antioxidant, has been proposed as a potential therapeutic agent against SARS-CoV-2 via down-regulation of ACE2 122, 123. Similarly, kurarinone, a prenylated flavonone isolated from the roots of Sophora flavescens, has been reported to inhibit infection of a human coronavirus, HCoV-OC43, in human lung fibroblast MRC-5 cells in a dose-dependent manner 124. Interestingly, the anti-viral activity of kurarinone is associated with its capacity to impair the virus-induced autophagic flux. Obviously more work is needed to test the effects of kurarinone on SARS-CoV-2 and in in vivo models.

### 5.2 Agents targeting the mTORC1-autophagy-pathway

It is well established that mTORC1 is the most important negative regulator of autophagy *via* its dual inhibitory effects at both the initiation stage (via ULK1 complex) and the degradation stage (via TEFB and lysosome 22, 125 (**Figure 1**). Also the catalytic inhibitors of mTORC1 (such as Torin1), but not the allosteric inhibitors (such as rapamycin), are known to activate the lysosomal function, partly *via* the mTORC1-TFEB signaling axis 126, 127. Rapamycin and its analogs (Rapalogs) have been well developed as immune-suppressors, anti-aging and as anti-cancer therapeutics 128, 129. At present, there is evidence suggesting that rapamycin and Rapalogs have therapeutic benefits in COVID-19, via multiple mechanisms: suppression of viral protein synthesis *via* the host and inhibition of the inflammatory response and cytokine storm 130, 131. Clinically, there are some observations supporting their potential therapeutic benefit in COVID-19. For instance, a case report from Australia found that a Rapalog, everolimus has mitigating roles in COVID-19 from organ transplant patients 132. Tuberous sclerosis complex (TSC) patients are clinically featured with benign tumors in multiple organs due to constitutive activation of mTORC1 and often treated with mTOC1 inhibitors. Interestingly, a cohort study in a TSC clinic in Italy revealed the similar trend: those patients receiving treatment with Rapalogs were found to have better clinical outcomes in COVID-19 133. However, in tissue culture and in immunologically naive animals, there is evidence that exposure to Rapalogs enhances vulnerability to SARS-CoV-2 infection 134. Such controversial results have raised questions about whether Rapalogs can be used as a treatment for Covid-19.

Metformin, a well-known AMPK activator and mTORC1 inhibitor, has also been reported to have therapeutic benefits in elderly COVID-19 patients at nursing homes in USA 135. According to a recent cell model, metformin pre-treatment resulted in further phosphorylation of AMPK and caused a ten-fold reduction of SARS-CoV-2 viral titers 136. Nevertheless, mTORC1 hyperactivation is associated to ACE2 overexpression, which is one of the mechanisms explaining the possible therapeutic benefit of mTORC1 inhibitors 137. At present, there are clinical trials ongoing to test the therapeutic efficacy of Rapalogs and metformin in treatment of COVID-19 (**Table 1**). More work is needed to understand the moleuclar mechanisms underlying their possible therapeutic effects of against COVID-19.

Virus entry *via* endocytic pathway involves both endosome and lysosome, with acidic pH in their lumen 10. The cytoplasmic cAMP pool produced by soluble adenylyl cyclase (sAC) promotes V-ATPase recruitment to endosomes/lysosomes and thus their acidification 138, 139. Therefore, targeting the sAC-specific cAMP pool could serve as a potential strategy to impair the endocytic pathway and to hinder the entry of the SARS-CoV-2 into the host cell 140. At present specific sAC inhibitors such as SQ22536 have been developed 141 and it remains to be tested whether such inhibitors would possess any anti-viral activity against SARS-CoV-2 in vivo.

In the above-mentioned studies, it remains to be tested whether activation of autophagy is implicated in the therapeutic activity of those rapalogs and metformin against COVID-19. AR12 is a derivative of celecoxib which lost its inhibitory effect on COX2 but instead inhibits the ATPase activity of multiple chaperone proteins, in particular GRP78 142. GRP78 acts as a sensor ER stress and plays a negative role in inhibiting autophagy and viral replication 143. A recent study has provided evidence linking the anti-viral function of AR12 against SARS-CoV-2 with activation of autophagy 64. In their study, the authors found that AR12 suppresses the production of infectious SARS-CoV-2 virions, probably *via* inhibiting the synthesis of the SARS-CoV-2 spike protein, a process related to AR12-mediated suppression of GRP78 and activation of autophagy 64.

Plitidepsin is a cyclic depsipeptide extracted from the ascidian *Aplidium albicans* and possess antitumor, antiviral and immunosuppressive activities *via* its potent inhibitory effect on eukaryotic translation elongation factor 1 alpha 1 (eEF1A1) 144. A very recent study reported that plitidepsin has potent preclinical efficacy against SARS-CoV-2 by targeting the host protein eEF1A, with a much higher potency than that of remdesivir 145. Treatment with plitidepsin was well-tolerated without any further hematological or cardiovascular toxicities, which supported plitidepsin as a potential antiviral drug in SARS-CoV-2 patients affected by immune deficiencies and hematological malignancies 146. Plitidepsin has completed one phase I clinical trial in patients with COVID-19, demonstrating that it has a favorable safety profile 147 (**Table 1**). Since the pharmacological effects of plitidepsin is also known to be linked to autophagy and lysosome 145, 148, its functional implication of the autophagy-lysosome pathway in the antiviral activity of plitidepsin against SARS-CoV-2 remains to be further tested.

## 6. Summary and perspectives

In summary, there is growing amount of evidence indicating the interplays between autophagy and SARS-CoV-2 and the implications of the autophagy-lysosome pathway in the pathogenicity of SARS-CoV-2 and the disease incurred COVID-19. So is autophagy a friend or a foe for SARS-CoV-2? Is the autophagy-lysosome pathway the legitimate target in the treatment of COVID-19? Unfortunately, it is still too early to provide an unequivocal answer at this stage, mainly based on the following facts: First, despite the extensive efforts in the past one year, most of the studies on autophagy and SARS-CoV-2 were not based on full and live SARS-CoV-2, instead using model CoVs such as MHVs or only using some components of SARS-CoV-2. This is largely due to the lack of necessary infrastructure (P3-level or BSL 3 lab) for most of the research laboratories in handling live SARS-CoV-2. Second, most studies were based on cell culture models and evidence from in vivo animal models with specific ATG deletion using live SARS-CoV-2 is still scarce, partly due to the low availability of proper animal models such as transgenic mouse model with expression of hACE2. Third, as the existing evidence demonstrates the possible implication of some key components of the autophagy-lysosome pathway, but not the whole autophagy process, it remains to be tested whether the other forms of autophagy such as CMA and microautophagy are involved. And fourth, so far, no randomized double-blinded large sample clinical trials have been conducted to test the therapeutic efficacy of lysosomotropic agents (except CQ/HCQ), so it is still too immature to conclude whether modulation of the autophagy-lysosome pathway is effective in the treatment of COVID-19.

To move forward, with the untamed spreading of SARS-CoV-2 across the globe, deeper understanding of the underlying molecular mechanisms of the pathogenicity of SARS-CoV-2 and development of the effective and specific therapeutics remain critically important and unfinished tasks. One strategy is to tackle the autophagy-lysosome pathway for this purpose. To overcome the pitfalls and limitations of the current studies, there are several lines of work needed. First, use transgenic mouse models with selective deletion of ATG genes and overexpression of hACE2 to mimic the SARS-CoV-2 infection in human. Second, repurpose of existing therapeutics based on their modulation effects on autophagy and lysosome should be a priority, since development of new drugs is much more costly and time-consuming. Last, a combinational therapy with existing anti-viral therapeutics such as remdesivir and CQ/HCQ should be included in more clinical trials to examine the therapeutic benefits.

## Figures and Tables

**Table 1:** Summary of interventional clinical trials using therapeutics targeting the autophagy-lysosome system in COVID-19

Therapeutic agents	Target molecules/organelles and mechanisms	Types/Phases	Status	Remarks
CQ/HCQ	Endosome/ Lysosome, inhibition of lysosomal acidification	Open-label non-randomized clinical trial Mostly double-blind, placebo-controlled, Phase 3 25 trials reached Phase 4.	17 Active, not recruiting 50 Completed 5 Enrolling by invitation 20 Not yet recruiting 37 Recruiting 6 Suspended 35 Terminated 1 Unknown status 34 Withdrawn	So far the most well-studied lysosomotropic agents for COVID-19, with hundreds of clinical trials all over the world.
Azithromycin	Endosome/ Lysosome, inhibition of lysosomal acidification	Open-label non-randomized clinical trial Mostly double-blind, placebo-controlled, Phase 2 and 3 Several trials reached Phase 4	7 Active, not recruiting 16 Completed 1 Enrolling by invitation 6 Not yet recruiting 22 Recruiting 4 Suspended 8 Terminated 13 Withdrawn	The published results were mostly from open-label non-randomized clinical trials, showing either Azithromycin alone or in combination with CQ/HCQ are effective in treatment of COVID-19.
Azithromycin + CQ/HCQ	Endosome/ Lysosome, inhibition of lysosomal acidification	Open-label non-randomized clinical trial Mostly double-blind, placebo-controlled, Phase 2 and 3	3 Active, not recruiting 6 Completed 1 Not yet recruiting 5 Recruiting 1 Terminated 7 Withdrawn	
GNS561	Endosome/ Lysosome, inhibition of PPT1 and cathepsin activity in lysosome	Open-label, controlled, randomized phase 2	1 Recruiting	Results pending
Artemisinin/ Artesunate	Endosome/ Lysosome, inhibition of autophagosome turnover	Double-blind, Placebo-Controlled, Phase 2	4 Completed 2 Not yet recruiting 4 Recruiting 2 Terminated	Results pending
Pyronaridine-Artesunate (PA)	Endosome/ Lysosome, inhibition of autophagosome turnover	Multi-center, Randomized, Double-blind, Parallel, Placebo-Controlled, Phase 2	2 Completed 2 Recruiting	Results pending
Plitidepsin	Inhibition of eEF1A2 and possibly autophagy and lysosome functions	Multicenter, Randomized, Parallel and Proof of Concept Study	1 Completed 2 Recruiting	Completed in Dec 2020, with 46 patients enrolled, which shows Plitidepsin has a favorable safety profile in patients with COVID-19
Rapamycin	mTORC1 , allosteric inhibition of the kinase activity of mTORC1	Randmized, double blind, placebo-controlled phase 2	1 Withdrawn	Results pending
Metformin	AMPK, activation of AMPK kinase activity	Multicenter, Prospective, Adaptive, Double-blind, Randomized, Placebo-controlled phase 2	1 Not yet recruiting 1 Recruiting 1 Completed 1 Withdrawn	Results pending

Data were obtained from the 7528 clinical trial portal at NIH (https://www.clinicaltrials.gov/) available by Feb 15, 2022.

## Abbreviations

AMPK: AMP protein kinase
ATG: Autophagy-related genes
CQ: chloroquine
CoVs: Coronaviruses
COVID-19: coronavirus disease of 2019
HCQ: hydroxychloroquine
mTORC1: mammalian target of rapamycin complex 1
SARS-CoV-2: severe acute respiratory syndrome coronavirus 2
